# Human cases of lymphocytic choriomeningitis virus (LCMV) infections in Hungary

**DOI:** 10.1007/s00705-023-05905-4

**Published:** 2023-10-19

**Authors:** Péter Pankovics, Arnold Nagy, Zoltán Nyul, Annamária Juhász, Károly Takáts, Ákos Boros, Gábor Reuter

**Affiliations:** 1 Department of Medical Microbiology and Immunology Medical School, University of Pécs, Szigeti út 12, Pécs, H-7624 Hungary; 2 Department of Paediatrics, Medical School, Medical School, University of Pécs, Pécs, Hungary; 3 Department of Neurology, Medical School, University of Pécs, Pécs, Hungary

## Abstract

Lymphocytic choriomeningitis (LCM) is a “neglected” rodent-borne viral zoonotic disease caused by lymphocytic choriomeningitis virus (LCMV) (family *Arenaviridae*). The aim of this retrospective clinical and laboratory study was to detect LCMV RNA, using RT-PCR, in cerebrospinal fluid samples collected from patients with central nervous system (CNS) infections of unknown aetiology from over a 12-year period in Hungary. Between 2009 and 2020, a total of 74 cerebrospinal fluid samples were tested using an in-house LCMV-specific RT-PCR-based method at the Department of Medical Microbiology and Immunology, University of Pécs. The mean age of the 74 patients included in our study was 24 years (min. 5, max. 74), with a predominance of men (44 [59.5%]; women, 30 [40.5%]). Two (2.7%) cerebrospinal fluid samples were found to be positive for LCMV RNA by RT-PCR and sequencing. The first LCMV case was a 5-year-old preschool boy who had a hamster bite on his left-hand finger, and the second LCMV case was a 74-year-old man who was living in a village and had incipient dementia and a previous permanent functional CNS impairment. The two detected LCMV strains (MW558451 and OM648933) from the year 2020 belonged to two different genetic lineages (I and II). These two cases of CNS inflammation of unknown origin represent the first published human LCMV infections confirmed by molecular methods in Hungary.

**Supplementary Information:**

The online version contains supplementary material available at 10.1007/s00705-023-05905-4.

In most cases, the aetiological background of acute inflammation of the central nervous system (CNS) remains unknown [1]. Lymphocytic choriomeningitis virus (LCMV) (genus *Mammarenavirus*, family *Arenaviridae*) is one of the causative agents of acute (meningo)encephalitis in humans worldwide, and until 2023 [2], it was the only known arenavirus in Europe [3, 4]. LCMV is an enveloped virus with a segmented, single-stranded ambisense RNA genome. LCMVs are classified into four different genetic lineages based on phylogenetic analysis of the S segment [5].

Lymphocytic choriomeningitis (LCM), caused by LCMV, is a rodent-borne viral zoonotic infection whose epidemiological, clinical, and laboratory diagnostic aspects have not been extensively studied. The main reservoir of LCMV is the house mouse (*Mus musculus*), but other rodents (e.g., hamsters) can also serve as hosts [5, 6]. Human-to-human transmission has not yet been confirmed, but transplacental transmission and transmission through infected organs have been reported [7, 8]. LCMV infection in immunocompetent individuals may be asymptomatic or present as a nonspecific [7], self-limited febrile disease [5, 9]. However, the illness can progress to meningitis, meningoencephalitis with symptoms of headache, vomiting, photophobia, and nuchal rigidity, or severe neurological birth defects [5, 10]. The initial laboratory abnormalities may include leukopenia, thrombocytopenia, and mild elevation of liver enzymes, but during the second phase of the illness, affecting the CNS, there is a characteristic increase of white blood cells and mild elevation of cerebrospinal fluid (CSF) proteins in the CSF [5, 10]. No specific treatment or antiviral drug is available [11]. The seroprevalence of the virus is variable but can be as high as 47% in populations exposed to rodents [5]. The LCMV can be detected by serological (ELISA)-, immunofluorescence assay (IFA)-, and PCR-based methods [7].

LCM is underdiagnosed in both clinical and laboratory practice. The aim of this retrospective clinical and laboratory study was to detect LCMV viral RNA, using the RT-PCR method, in cerebrospinal fluid samples collected from CNS infections of unknown aetiology over a 12-year period, between 2009 and 2020, in Hungary.

A total of 74 blood and cerebrospinal fluid samples were collected between 2009 and 2020 from hospitalized patients in Baranya, Tolna, and Pest counties in Hungary with a clinical diagnosis of encephalitis and tested simultaneously by routine serological and molecular diagnostic methods for human herpesvirus types 1 and 2 (HHV1/2). Blood samples were screened using HHV1/2 IgM/IgG ELISA kits (HSV-IgM/IgG ELISA, Dia.Pro, Italy), and total nucleic acids were extracted from blood and cerebrospinal fluid samples using a High Pure Viral Nucleic Acid Kit (Roche, Switzerland). Samples were tested prospectively by PCR for HHV1/2 [12] and retrospectively using an in-house RT seminested PCR assay, using the primers LCMV-F1 (5'-ACNTGGCAYATGCAYAA-3'), LCMV-F2 (5'-AGYCTHATTGAYATGGG-3'), and LCMV-R (5'-ACYTCYTCNCCCCANACATA-3'), which were designed in this study for detection of the LCMV L segment (RdRp) (Supplementary Table S1.). Longer nucleotide sequences of the LCMV L segments were determined using sequence-specific reverse/forward primer sets and the 5’RACE technique [13]. PCR products were subjected to agarose gel electrophoresis and then sequenced directly using an automated sequencer (3500 Genetic Analyzer, Applied Biosystems, Japan).

Of the 74 sample pairs, 61 were from the University of Pécs, mostly from the Department of Paediatrics (N = 38) and the Department of Neurology (N = 16). Seven sample pairs were from county (Szekszárd, N = 6) or town (Szigetvár, N = 1) hospitals, and the other six were from the Hospital of Szent László, Budapest. The average age of the patients was 24 years (min. 5, max. 74) with a predominance of men (44 [59.5%]; women: 30 [40.5%]). Two (2.7%) cerebrospinal fluid samples tested positive for LCMV RNA by RT-PCR and sequencing (Fig. 1).

**Fig. 1:**
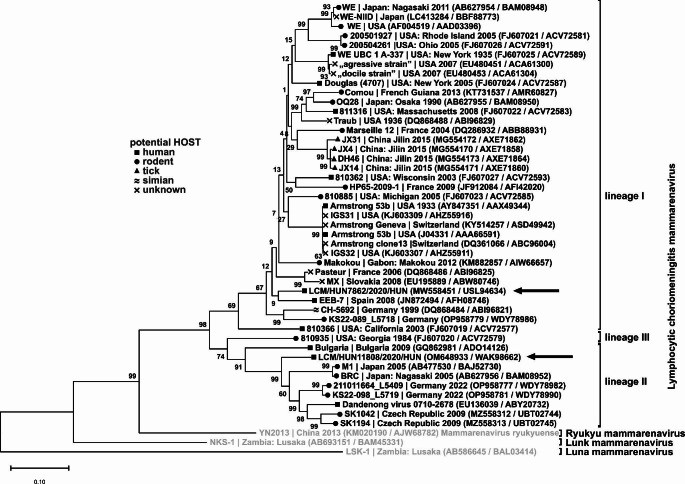
Phylogenetic analysis of lymphocytic choriomeningitis virus (LCMV) based on 220-amino-acid-long partial RdRp sequences encoded by the L segment. Sequences were aligned using MAFFT (Multiple Alignment using Fast Fourier Transform), and the alignment was analysed by the maximum-likelihood method based on the Jones-Taylor-Thornton (JTT) model (Uniform rate/Use all sites) in MEGA11 with 1000 bootstrap replicates. Viruses from this study are indicated by black arrows

Case 1: A 5-year-old preschool boy had a hamster bite on his left-hand finger two months before symptoms appeared on May 12, 2020 (Fig. 2). The accident happened in a nursery, where the pet was brought in for petting. The child presented to the Emergency Department with fever (> 38.5°C) lasting for 5 days, loss of appetite, lethargy, weakness, and repeated vomiting. On the day after hospital admission, his vomiting worsened, and when he also began to experience neck stiffness, he was transferred to the intensive care unit (ICU). The detected LCMV strain (HUN7862/2020/HUN, MW558451) belongs to genetic lineage I and shows 89/95% nucleotide/amino acid sequence identity to the corresponding region of the LCMV strain JX31/China Jilin (MG554172), which was detected in a tick in China [14] (Fig. 1).

**Fig. 2:**
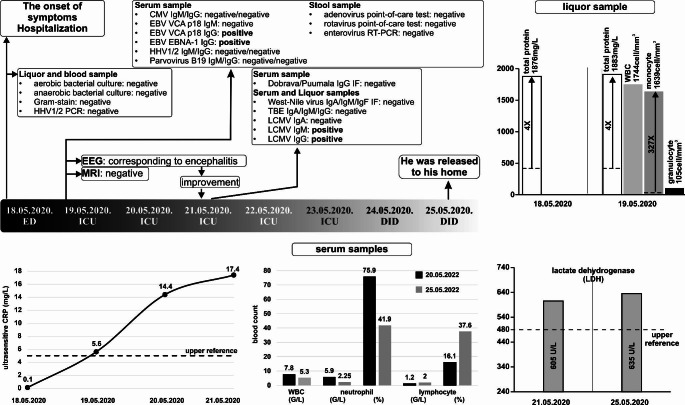
The case history of the 5-year-old child. An electroencephalography (EEG) test showed patterns characteristic of encephalitis, which was not confirmed by MRI. Acyclovir treatment was started immediately at admission. Routine microbiological tests of cerebrospinal fluid, blood (for aerobic, anaerobic bacteria, fungi, HHV1/2, CMV, EBV, and parvovirus B19) and stool samples (adenovirus, rotavirus, and enterovirus) did not reveal the presence of these pathogens. Further viral serology tests of blood and cerebrospinal fluid samples for hantaviruses, West Nile virus, and tick-borne encephalitis virus infections were negative, but positive results for LCMV IgM and IgG were obtained on May 21, 2020 (National Centre for Epidemiology, Budapest). Elevated C-reactive protein (up to 17.4 mg/L), lactate dehydrogenase (up to 635 U/L; normal range, 240–480 U/L), and a change in the percentage distribution of neutrophil granulocytes (-Δ34%) and lymphocytes (+Δ21.5%) was observed in the serum samples. In the cerebrospinal fluid, 4 times more total protein (1,883 mg/L; upper reference, < 450mg/L), white blood cells (WBC, 1,744 cells/mm^3^), and 327 times more mononuclear (1,639 cells /mm^3^; upper reference, < 5 cells /mm^3^) and granulocyte cells (105 cells /mm^3^, normally intact) were measured. His condition improved, and he was treated at the Department of Infectious Diseases (DID) on May 24, 2020, and was released healthy to his home on May 25, 2020. ED, emergency department; ICU, intensive care unit

Case 2: A 74-year-old man who was living in a village had incipient dementia and a previous permanent functional CNS impairment (vascular encephalopathy, ischemia) because of a stroke. He was transported on Oct 8, 2020 to the emergency department with confusion, weakness, fatigue, fever, aphasia, and difficulty in moving his left arm (Fig. 3). Later he had no fever, but psychomotorically, he was restless and began to have convulsions. The detected LCMV strain (HUN11808/2020/HUN, OM648933) belongs to the genetic lineage II and shows 82/90% nucleotide/amino acid sequence identity to the corresponding region of LCMV strain SK1194 (MZ558313) detected in mice in the Czech Republic [15] (Fig. 1).

**Fig. 3:**
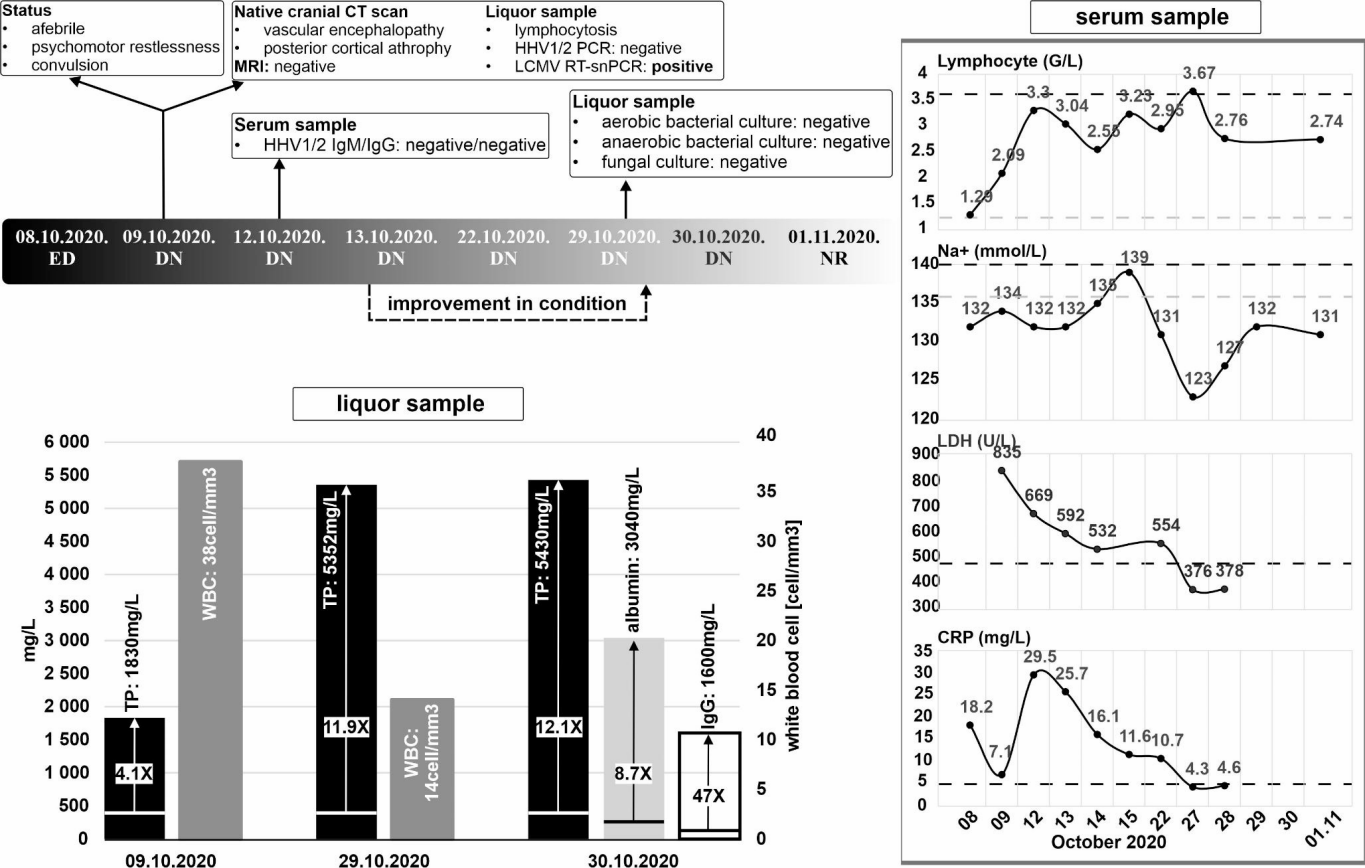
The case history of the 74-year-old man. No change confirming his acute deterioration was detected by a native cranial CT-scan and MRI. The patient was admitted to the Department of Neurology (DN), and based on the symptoms, acyclovir treatment was started immediately. A PCR test for HHV1/2 performed on cerebrospinal fluid was negative. A serological ELISA test for HHV1/2 in a serum sample was negative, and routine microbiological tests of the cerebrospinal fluid sample did not reveal pathogens for aerobic or anaerobic bacteria or fungi. The patient’s biomarkers in cerebrospinal fluid and serum showed significant deviations from normal. The initial total protein (TP) value in a cerebrospinal fluid sample was 1,830 mg/L, which was 4.1 times higher than the normal reference value, and it increased to 12.1 times higher on Oct 30, 2020. Moreover, 8.7 times higher albumin and 47 times higher immunoglobulin G concentrations than the normal value were measured in the cerebrospinal fluid. The white blood cell (WBC) count decreased from 38 cells /mm^3^ to 14 cells /mm^3^ between Oct 29 and Oct 30, 2020. Analysis of serum samples over time showed an increased lymphocyte count, elevated lactate dehydrogenase (LDH), C-reactive protein (CRP), and reduced sodium concentration. Dashed lines indicate the lower and upper reference ranges. After the patient’s condition improved, he was transferred on Oct 13, 2020, to the neurorehabilitation department (ND)

The nucleotide and amino acid identity between the two Hungarian LCMV strains in a portion of the L segment (RdRp) was 85% and 91%, respectively.

LCMV is a rodent-borne zoonotic pathogen [5, 6], but the infection has been largely neglected, both in Hungary and internationally. In Hungary, a total of 1,424 cases of acute encephalitis were reported between 2009 and 2020, 622 of which (43.7%) were of unknown aetiology based on laboratory testing. During this period, two LCMV cases were confirmed using RT-PCR among 74 patients with suspected CNS infection, suggesting the aetiology of the disease. Interestingly, HHV1/2 and LCMV viral nucleic acids were detected at equal frequency (2 out of 2 cases), which can certainly draw attention to the important but hidden prevalence of LCMV.

In our study, a young child and an elderly man tested positive for LCMV, which may indicate a wide age range for susceptibility to the disease, especially in potentially age-related immunologically weak groups. The possible source of an infection is always an important question. In case 1, there was contact with a hamster and even an animal bite in the patient’s medical history during the period before the disease began. In case 2, repeated (hetero)anamnesis was no longer possible due to the patient’s condition; however, he lived in a rural environment, and his family also raised animals. It can be assumed that, in a rural environment where feed is stored, rodents are common. The man’s underlying medical condition presumably also made it more difficult to maintain good hygiene. The clinical suspicion of LCM may be facilitated by an adequate anamnesis (e.g., animal contact), clinical symptoms of CNS inflammation, and integrated interpretation of the results of laboratory chemistry tests. In the presented cases, the clinical diagnosis of “viral encephalitis” was already made during the patients’ care based on the clinical picture and, partly, the results of instrumental investigations. In case 2, the history of stroke made it difficult to interpret clinical symptoms. Laboratory chemistry tests of cerebrospinal fluid and sera showed markedly elevated levels of biomarkers, including total protein, which reached extreme values in case 2.

The two LCMV isolates from Baranya County obtained in 2020 belonged to two different genetic lineages (I and II), and therefore, a common source of infection can be excluded. The habitat zones of the main host species, *Mus musculus* and *M. domesticus*, potentially associated with different genetic lineages of LCMV, run through Central and Eastern Europe, including Hungary [15]. Further specific research is needed to investigate the diversity of the circulating genetic lineages of LCMV that are endemically present in wild (and pet) animals in Hungary. The fact that both human cases occurred in 2020 is an interesting observation, raising the question of whether this is a coincidence or there is a significant but unknown environmental factor in the background.

In our study, we found evidence of two cases of overlooked LCM in patients with a CNS inflammation of unknown origin, which represent the first human LCMV infections confirmed by molecular methods and published in Hungary. Our results confirm that the pathogenic role of LCMV in encephalitis/meningitis should be considered in clinical practice, and clinical microbiological laboratories must have appropriate diagnostic tools to confirm the infection. Until recently, LCMV was the only known endemic arenavirus in Europe. In the light of the second mammarenavirus in Europe serendipitously discovered using the “LCMV primers” originally designed for this study [2], our knowledge of the biology of arenaviruses, as well as the pathogenicity for humans, significance, and genetic diversity of LCMV are still far from complete.

### Electronic Supplementary Material

Below is the link to the electronic supplementary material.

Supplementary Material 1
