# Supplementary material for: Human cases of lymphocytic choriomeningitis virus (LCMV) infections in Hungary
Source: Arch Virol. 2023 Oct 19;168(11):275. doi: 10.1007/s00705-023-05905-4 (PMC10584706; doi:10.1007/s00705-023-05905-4)
Supplement: Supplementary file 1 — Supplementary Material 1 [file 705_2023_5905_MOESM1_ESM.docx]

**Supplementary Table S1** Primer names, sequences and application in this study

| Primer | Sequence | Location | Source | Use | Reagents (final concentration) | Cycling protocol |
| --- | --- | --- | --- | --- | --- | --- |
| LCMV-F1 | 5'-ACNTGGCAYATGCAYAA-3' | 3670* | in-house | cDNA synthesis / PCR1 | 10X DreamTaq Buffer (1X, Thermo Fisher), dNTP mix (0.4 mM, Thermo Fisher), LCMV-F1 (0.8 µM), ribonuclease inhibitor (10 U, Promega), M-MLV-RT reverse transcriptase (50 U, Promega) 2.5 µl RNA and Nuclease-free water up to 25 µl final volume | 42°C for 55 min, 85°C for 5 min, then 4°C for until use |
| LCMV-R | 5'-ACYTCYTCNCCCCCANACATA-3' | 4140* |  | PCR1/PCR2 | 10X DreamTaq Buffer (1X), LCMV-F1 (0.8µM), LCMV-R (0.8µM), DreamTaq DNA polymerase (5U) and Nuclease-free water up to 25 µl final volume and used as one step with RT reaction. | 95°C for 2 min, 34 cycles of 95°C for 30 sec, 50°C for 20 sec, 72°C for 30 sec, final elongation 72°C for 5 min, and 4°C for 5 min |
| LCMV-F2 | 5'-AGYCTHATTGAYATGGG-3' | 3840* |  | PCR2 | 10X DreamTaq Buffer (1X), dNTP mix (0.4 mM), LCMV-F2 (0.8 µM), LCMV-R (0.8 µM), DreamTaq DNA polymerase (5 U), 2 µl PCR1 template and Nuclease-free water up to 25 µl final volume. | 95°C for 2 min, 34 cycles of 95°C for 30 sec, 48°C for 20 sec, 72°C for 20 sec, final elongation 72°C for 5 min, and 4°C for 5 min |
| * The positions of the primers are based on the LCMV reference genotype strain Armstrong 53b (accession number NC_004291). Base analogues: N = (A, C, G, or T), Y = (C, or T), H = (A, C, or T). Primer pairs were designed based on the conservative regions of the RdRp gene (L segment) of the reference and all available LCMV strains (accession numbers: AB477530, AB627954, AB627955, AB627956, AF004519, DQ286932, DQ361066, DQ868484, DQ868486, DQ868488, EU195889, EU480451, EU480453, FJ607019, FJ607020, FJ607021, FJ607022, FJ607023, FJ607024, FJ607025, FJ607026, FJ607027, GQ862981, JF912084, JN872494, KJ603307, KJ603309, KT731537, LC413284, MG554170, MG554171, MG554172, MG554173). | | | | | | |
